# A Japan nationwide web-based survey of patient preference for renal denervation for hypertension treatment

**DOI:** 10.1038/s41440-021-00760-9

**Published:** 2021-10-17

**Authors:** Kazuomi Kario, Hideaki Kagitani, Shoko Hayashi, Satsuki Hanamura, Keisuke Ozawa, Hiroshi Kanegae

**Affiliations:** 1 Division of Cardiovascular Medicine, Department of Medicine, Jichi Medical University School of Medicine, Tochigi, Japan; 2 Clinical Development Department, Terumo Corporation, Tokyo, Japan

**Keywords:** Hypertension, Patient preference, Renal denervation, Blood pressure

## Abstract

Renal denervation is a potential alternative to antihypertensive drug therapy. However, data on patient preference for this treatment option are limited and there are no data specifically from Asian patients. This study evaluated patient preference for renal denervation in patients with hypertension from Japan. Patients were a subset of those who participated in a March 2020 online electronic survey of patients with hypertension who had regularly visited medical institutions for treatment, were receiving antihypertensive drug therapy and had home blood pressure recordings available. The survey included a question about patient preference for treatment with renal denervation. A total of 2,392 patients were included (66% male, mean age 59.8 ± 11.6 years, mean duration of hypertension 11.4 ± 9.5 years). Preference for renal denervation was expressed by 755 patients (31.6%), and was higher in males than in females, in younger compared with older patients, in those with higher versus lower blood pressure, in patients who were less adherent versus more adherent to antihypertensive drug therapy, and in those who did rather than did not have antihypertensive drug-related side effects. Significant predictors of preference for renal denervation on logistic regression analysis were younger patient age, male sex, higher home or office systolic blood pressure, poor antihypertensive drug adherence, the presence of heart failure, and the presence of side effects during treatment with antihypertensive drugs. Overall, a relevant proportion of Japanese patients with hypertension expressed a preference for renal denervation. This should be taken into account when making shared decisions about antihypertensive drug therapy.

## Introduction

An estimated 1.13 billion people worldwide have hypertension [1]. Despite a growing number of therapeutic options for hypertension, less than 20% of patients globally who are being treated for high blood pressure (BP) achieve BP control [1], something that has been described as the “hypertension paradox” [2].

In Japan, there were 43 million individuals with hypertension in 2017 [3]. Of these, only half were receiving treatment, and an even smaller proportion (just over one-quarter) achieved BP control based on a target of <140/90 mmHg [4]. The lowering of BP targets in the latest Japanese [3] and American [5] guidelines, to 130/80 mmHg for most patients with hypertension, means that the proportion of patients with adequately controlled BP is even lower [6]. Effective control of hypertension is essential to reduce cardiovascular risk [7, 8].

Based on the important pathophysiological role of the sympathetic nervous system in hypertension [9], catheter-based renal denervation (RDN) has been developed as a new treatment approach to reducing BP (Fig. S1) [10]. Over the last ten years, data from clinical trials of RDN have been mixed [11–13]. However, more recent studies with second-generation ultrasound- and radiofrequency-based RDN devices have produced promising results [14–19].

To date, there is a relative lack of data on the effects of renal denervation in Asian patients with hypertension [20]. This is partly due to the early termination of the SYMPLICITY-HTN-JAPAN [21] study after one of the early trials of radiofrequency RDN, the SYMPLICITY HTN-3 study, did not show a significant reduction in systolic BP (SBP) in patients with resistant hypertension treated with RDN compared with the sham control group at 6-month follow-up [11]. Nevertheless, RDN has the potential to be an useful option for Asian patients with hypertension, who have a specific disease phenotype that includes a stronger association between BP and cardiovascular disease compared with Western populations, and high salt sensitivity [22]. In addition, Asian patients appear to be more sensitive to beta-blockers [23], suggesting that RDN (as another treatment that blocks the sympathetic nervous system) may be an appropriate and effective therapy [24].

In Germany, a significant proportion of patients with elevated BP stated that they would prefer catheter-based RDN compared with ongoing antihypertensive drug therapy [25]. The current study was designed to evaluate patient preference for RDN in patients with hypertension from Japan.

## Methods

### Study design

An electronic survey of patients with hypertension registered with the marketing research firm Macromill Carenet was conducted in March 2020 to collect information on hypertensive outpatients in Japan (UMIN000039726). All data provided by the subjects online was anonymized and stored in a database. The study received ethical approval (approval number: CR19-R049), and all patients provided informed consent prior to completing the online survey.

### Study population

Participants had regularly visited medical institutions for the treatment of hypertension with antihypertensive drug therapy. Those aged <18 or >80 years at the time of survey completion were excluded. This study included patients being treated with antihypertensive drug therapy who had home BP recordings available.

### Survey

The survey collected data on participant age, sex, area of residence, comorbidities, frequency of clinic visits for hypertension management, antihypertensive drug classes prescribed, total number of antihypertensive drugs taken per day, and the most recent home and office BP values (see Supplementary Methods for full details). In addition, patients were asked about their preference for treatment with RDN based on which of the following responses they chose: “I don’t want to undergo RDN”; “I’d rather not undergo RDN”; “I’d rather undergo RDN”; and “I want to undergo RDN”. Patients who chose either of the last two responses were defined as having a preference for RDN.

### Statistical analyses

All statistical analyses were performed using SAS 9.4 (SAS Institute, North Carolina, USA). Categorical variables are described using frequencies and percentages, while continuous variables are reported as mean values with standard deviations (SD). Categorical variables were compared using the Chi-squared test and the unpaired t-test were used to compare continuous variables between groups. The Kruskal–Wallis test was used to test for significant differences between patient preference (ordinal scale) and both BP levels and the number of antihypertensive medications. Stepwise logistic regression analysis was used to identify the predictors of patient preference for RDN. Statistical significance was defined as a two-sided *p*-value of  < 0.05.

## Results

Of 4,107 patients who answered questions relating to preference for RDN, 2,392 had submitted home BP readings and were included in the current analysis. The majority of patients (66%) were male, mean age was 59.8 ± 11.6 years, and mean duration of hypertension was 11.4 ± 9.5 years (Table 1). Baseline mean office and morning home BP values were 135.7 ± 14.2/82.9 ± 11.1 mmHg and 134.6 ± 14.3/83.1 ± 11.5 mmHg, respectively (Table 1).

**Table 1:** Patient demographic and clinical characteristics at baseline

Characteristic	Patients (*n* = 2,392)
Age, years	59.8 ± 11.6
Male, *n* (%)	1,577 (65.9)
Body mass index, kg/m^2^	25.1 ± 4.4
Duration of hypertension, years	11.4 ± 9.5
Office blood pressure	
SBP, mmHg	135.7 ± 14.2
DBP, mmHg	82.9 ± 11.1
Uncontrolled SBP or DBP (≥130 or ≥80 mmHg), n (%)	1,964 (19.9)
Uncontrolled SBP or DBP (≥140 or ≥90 mmHg), n (%)	909 (57.9)
Morning home blood pressure	
SBP, mmHg	134.6 ± 14.3
DBP, mmHg	83.1 ± 11.5
Uncontrolled SBP or DBP (≥125 or ≥75 mmHg), *n* (%)	2,150 (89.9)
Uncontrolled SBP or DBP (≥135 or ≥85 mmHg), *n* (%)	1,364 (57.0)
Medical history, *n* (%)	
Diabetes mellitus	553 (23.1)
Cardiovascular disease	476 (19.9)
ASCVD	442 (18.5)
CAD	250 (10.5)
Stroke	196 (8.2)
Aortic aneurysm/dissection, PAD	123 (5.1)
Heart failure	133 (5.6)
Chronic kidney disease	166 (6.9)
Medical facility for hypertension treatment, *n* (%)	
Medical university hospital	119 (5.0)
Hospital	651 (27.2)
Clinic	1,622 (67.8)
Antihypertensive therapy	
Number of antihypertensives, *n* (%)	
1	886 (37.0)
2	1,261 (52.7)
≥3	245 (10.2)
Time on antihypertensives, years	10.3 ± 8.8
Poor adherence^a^, *n* (%)	288 (12.0)
Side effects present^§^, *n* (%)	911 (38.1)

Values are mean ± standard deviation, or number of patients (%)

^a^Poor adherence was defined as missing at least one antihypertensive dose per week. ^§^Side effects attributable to antihypertensive medication

*ASCVD* atherosclerotic cardiovascular disease, *CAD* coronary artery disease, *DBP* diastolic blood pressure, *PAD* peripheral artery disease, *SBP* systolic blood pressure

### Patient preference for RDN

**Fig. 1:**
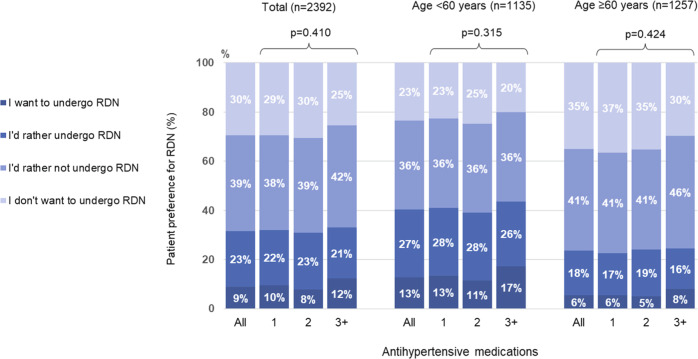
Relationship between patient preference for renal denervation (RDN) and the number of antihypertensive medications in patients aged < 60 years or ≥60 years

Overall, 215 respondents (9.0%) said that they wanted to undergo RDN and 540 (22.6%) said that they would rather undergo RDN, meaning that a total of 755 patients (31.6%) expressed a preference for RDN. Patient preference for RDN did not vary significantly by the number of antihypertensive agents being taken, but a higher proportion of younger versus older patients had a preference for RDN (Fig. 1).

**Fig. 2:**
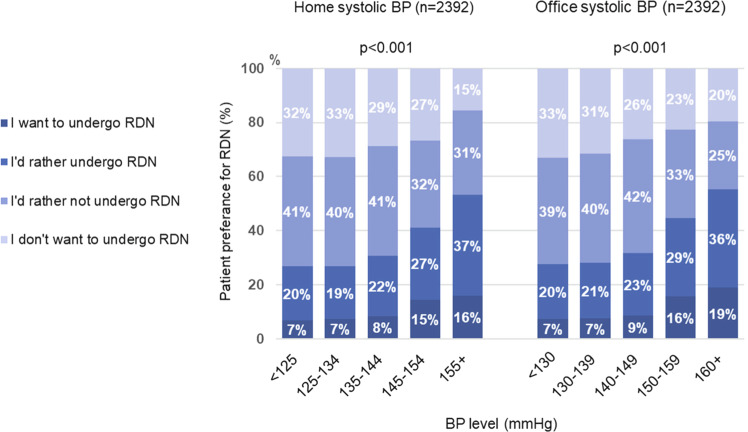
Relationship between patient preference for renal denervation (RDN) and levels of home and office systolic blood pressure (BP)

**Fig. 3:**
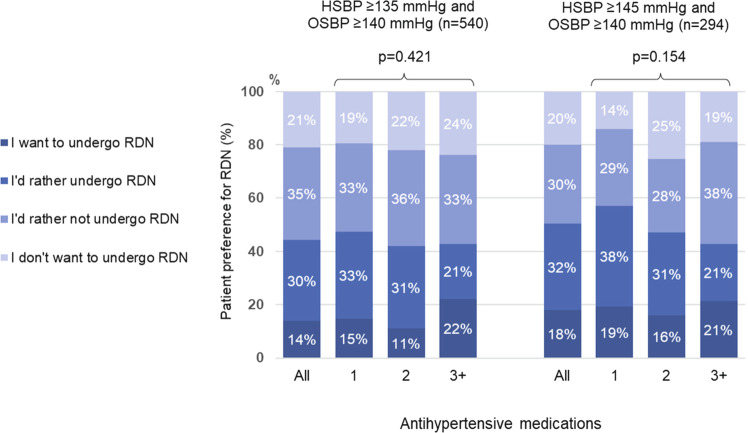
Relationship between patient preference for renal denervation (RDN) and the number of antihypertensive medications in patients with grade I or grade II hypertension (as defined in the 2019 Japanese Society of Hypertension guidelines [3])

There were significant differences in patient preference for RDN between patient subgroups based on home and office SBP values (Fig. 2). The proportion of patients expressing a preference for RDN increased as both home and office SBP increased, being highest in those with office SBP ≥ 160 mmHg or home SBP ≥ 155 mmHg (Fig. 2). In patients grade I or II hypertension [3], almost half of patients expressed a preference for RDN (Fig. 3).

There was also a significant relationship between adherence to antihypertensive medication and preference for RDN; patients who were less adherent to pharmacological antihypertensive therapy were significantly more likely to express a preference for RDN (Table 2).

**Table 2:** Adherence to prescribed antihypertensive medication and patient preference for renal denervation

Adherence	Number of patients (%)	Patient preference for RDN^a^, n (%)	*p*-value^b^
Always take antihypertensives	1,582 (66.1)	456 (28.8)	*p* < 0.001
Frequency of not taking antihypertensives:			
Once per month	370 (15.5)	116 (31.4)	
Once every 2 weeks	152 (6.4)	57 (37.5)	
Once a week	156 (6.5)	63 (40.4)	
Once every 2 days	49 (2.0)	23 (46.9)	
Don’t take every day	83 (3.5)	40 (48.2)	

^a^Patient preference for renal denervation was defined as a survey answer of “I want to undergo renal denervation” or “I would rather undergo renal denervation”

^b^Chi-square test

Significant predictors of preference for RDN on logistic regression analysis were younger patient age, male sex, higher home or office SBP, poor antihypertensive drug adherence, the presence of heart failure, and presence of side effects during treatment with antihypertensive drugs (Table 3).

**Table 3:** Determinants of patient preference for renal denervation

Variable	*N*	Patient preference^a^, *n* (%)	Univariate analysis		Logistic regression analysis	
			Crude OR (95% CI)	Crude *p*-value	Adjusted OR (95% CI)	Adjusted *p*-value
Age, years						
≤49	552	250 (45.3)	3.23 (2.51–4.16)	<0.001	2.99 (2.29–3.09)	<0.001
50–59	583	207 (35.5)	2.15 (1.66–2.77)	<0.001	2.24 (1.72–2.92)	<0.001
60–69	605	165 (27.3)	1.46 (1.13–1.90)	0.004	1.51 (1.15–1.98)	0.003
≥70	652	133 (20.4)	reference	—	reference	—
Sex						
Male	1,577	553 (35.1)	1.64 (1.36–1.98)	<0.001	1.71 (1.40–2.08)	<0.001
Female	815	202 (24.8)	reference	—	reference	—
Office SBP, mmHg						
≥160	159	88 (55.4)	3.26 (2.28–4.64)	<0.001	1.71 (1.16–2.53)	0.007
150–159	173	77 (44.5)	2.11 (1.49–2.97)	<0.001	1.63 (1.15–2.29)	0.006
140–149	430	136 (31.6)	1.21 (0.93–1.58)	0.147	*n.s*.	—
130–139	941	264 (28.2)	1.02 (0.82–1.28)	0.831	*n.s*.	—
≤129	689	190 (27.6)	reference	—	reference	—
Home SBP, mmHg						
≥155	201	107 (53.2)	3.11 (2.22–4.36)	<0.001	1.65 (1.16–2.35)	0.006
145–154	255	105 (41.2)	1.91 (1.40–2.62)	<0.001	1.42 (1.06–1.89)	0.018
135–144	622	191 (30.7)	1.21 (0.94–1.57)	0.144	*n.s*.	—
125–134	784	210 (26.8)	1.02 (0.82–1.28)	0.831	*n.s*.	—
≤124	530	142 (26.8)	reference	—	reference	—
Comorbidity						
Diabetes mellitus						
Present	553	198 (35.8)	1.28 (1.05–1.57)	0.014	*n.s*.	—
Absent	1,839	557 (30.3)	reference	—	reference	—
CAD						
Present	250	94 (37.6)	1.35 (1.03–1.77)	0.030	*n.s*.	—
Absent	2,142	661 (30.9)	reference	—	reference	—
Stroke						
Present	196	81 (41.3)	1.59 (1.18–2.14)	0.002	*n.s*.	—
Absent	2,196	674 (30.7)	reference	—	reference	—
Aortic aneurysm/dissection, PAD						
Present	123	51 (41.5)	1.57 (1.09–2.28)	0.015	*n.s*.	—
Absent	2,269	704 (31.0)	reference	—	reference	—
Heart failure						
Present	133	63 (47.4)	2.04 (1.43–2.90)	<0.001	1.51 (1.02–2.22)	0.039
Absent	2,259	692 (30.6)	reference	—	reference	—
Chronic kidney disease						
Present	166	64 (38.6)	1.39 (1.01–1.93)	0.045	*n.s*.	—
Absent	2,226	691 (31.0)	reference	—	reference	—
Adherence^**b**^						
Poor	288	126 (43.8)	1.82 (1.42–2.34)	<0.001	1.39 (1.06–1.82)	0.017
Good	2,104	629 (29.9)	reference	—	reference	—
Side effects^§^						
Present	911	375 (41.2)	2.03 (1.70–2.42)	<0.001	1.74 (1.44–2.09)	<0.001
Absent	1,481	390 (25.7)	reference	—	reference	—

*CAD* coronary artery disease, *CI* confidence interval, *n.s*. not significant, OR odds ratio, *PAD* peripheral artery disease, *SBP* systolic blood pressure

^a^Patient preference for renal denervation was defined as a survey answer of “I want to undergo renal denervation” or “I would rather undergo renal denervation”

^b^Poor adherence was defined as missing at least one antihypertensive dose per week. ^§^Side effects attributable to antihypertensive medication

Specific side effects that were significantly associated with a preference for RDN on logistic regression analysis were dizziness, frequent urination, palpitation/tachycardia, dry mouth, headache, hot flashes (women) and sexual dysfunction (men) (Table S2). By far the most common source of information about hypertension and a decision to undergo RDN was the patient’s physician (Fig. S2)

The expected reduction in BP after RDN was ≥5 mmHg in 5.5% of patients, ≥10 mmHg in 18.0% of patients, ≥15 mmHg in 12.8% of patients, ≥20 mmHg in 17.9% of patients, and ≥30 mmHg in 10.3% of patients (the remaining patients did not want RDN) (Fig. S3).

## Discussion

**Fig. 4:**
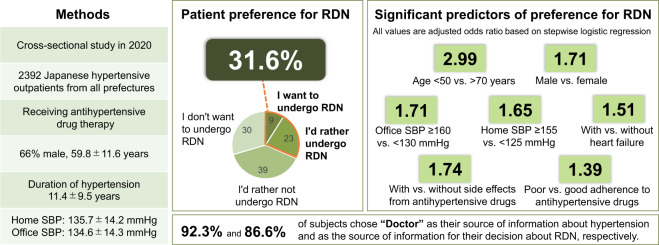
Graphical Abstract: A relevant proportion of patients with hypertension expressed a preference for renal denervation. This should be taken into account when making shared decisions about antihypertensive drug therapy

This is the first nationwide survey to investigate patient preference for RDN in Japanese patients with hypertension. Nearly one-third of the patients surveyed expressed a preference for RDN, and younger patients were more likely to prefer RDN than older patients. Those with more severe hypertension based on either office or home BP readings were also more likely to state a preference for RDN, with over 50% of subjects in the highest office and home BP categories preferring to undergo RDN. Both adherence to antihypertensive medication and the occurrence of drug-related side effects increased the number of patients stating a preference for RDN. For example, those with side effects during antihypertensive therapy were more than 1.7 times more likely to prefer RDN compared to patients without side effects. In terms of comorbidities, only the presence of heart failure was a significant predictor of preference for RDN (Fig. 4).

For patients with hypertension already on drug therapy, the proportion stating that they would prefer treatment with RDN in the current study (31.6%) was slightly higher than that in a similar study conducted in Germany (28.2%) [25]. This may reflect the greater body of information about RDN available in the two-year period between the timing of the German study and our survey in Japan. Other potential reasons for the difference in preference rates are the younger age and higher proportion of males in our study population, because younger age and male sex were significant independent predictors of a preference for RDN on logistic regression analysis in our study. Increasing age was also associated with decreasing RDN preference in both the German study [25] and an analysis from Taiwan [26]. It is possible that younger patients may be more motivated than older individuals to avoid the need for long-term antihypertensive therapy and regular physician visits. Expectations about the magnitude of BP reduction that would be associated with use of RDN were equivalent between the German survey and our research conducted in Japan (Fig. S3).

Looking at baseline BP levels, increasing office SBP and home SBP were related to higher rates of patient preference for RDN (Fig. 2). The proportion of patients expressing a preference for RDN was highest in those with office SBP ≥ 160 mmHg or home SBP ≥ 155 mmHg. In these groups, 55% and 53% of patients, respectively, said that they wanted or preferred to undergo RDN. This is consistent with the findings of a small survey conducted in Taiwan (*n* = 46), which showed that a higher proportion of patients with resistant hypertension were more likely to choose RDN than those without resistant hypertension (55.6% vs. 28.0%, respectively) [27]. In contrast, there was no relationship between current BP level and willingness to consider treatment with RDN in an analysis of data from market research studies conducted in Europe and the United States [28].

The current study from Japan is the first to include both home and office BP in assessments of patient preference for RDN. Out-of-office BP measurements are increasingly being recognized as important in the diagnosis and management of hypertension [3]. Home BP is an important component of out-of-office BP measurement and has been shown to be closely associated with cardiovascular risk [29–31]. Therefore, home BP is an important measurement for all patients with hypertension.

This survey showed that heart failure was the only comorbidity that was significantly associated with a preference for RDN. No specific information on the presence of heart failure was reported in the German survey [25]. However, combined data from Europe and the US showed that a significantly higher proportion of patients with comorbidities were willing to consider RDN rather than antihypertensive drug therapy [28]. The observed significant relationship between the presence of heart failure and preference for RDN in patients with hypertension in our survey likely reflects the symptomatic nature of heart failure and the desire for symptom resolution, and perhaps also the potentially positive effects of RDN in patients with heart failure [32–37]. In addition, patients with comorbidities may be more aware of the negative effects of hypertension on cardiovascular risk and renal disease, making them more likely to be motivated to reduce their BP.

We found a link between poor adherence to antihypertensive medication and patient preference for RDN. The proportion of patients in our survey who said that they “always adhere to antihypertensives” was 66%, compared with approximately 80% in other surveys [25, 28]. It does seem logical that patients who struggle to adhere to antihypertensive medication regimens would prefer a treatment that does not appear to require regular drug taking. However, antihypertensive drug usage might still continue after RDN, and inconsistent adherence to prescribed drugs after the procedure has been reported, with frequent non-adherence to antihypertensive medication [18]. Nevertheless, should accumulating clinical trial data continue to show that RDN has consistent and durable effects on BP in patients with hypertension, it would have the advantage of not being dependent on daily actions by the patient.

Another potential limitation of antihypertensive drug therapy that is overcome by the use of RDN is drug-induced adverse events. In the current survey, 38% of patients reported side effects related to their antihypertensive medications, and patients with side effects were 1.7 times more likely to express a preference for RDN than those without side effects. These findings are consistent with several other surveys that have reported higher rates of preference for RDN in patients with antihypertensive-related adverse effects [25–28].

The fact that 87% of patients in our study stated that their doctor was the source of information they used to make a decision about RDN (Fig. S2) highlights the important role of physicians in educating and informing patients about their treatment options, as well as hypertension itself. The key role of physicians has also been highlighted in other studies of patient preference for RDN [25, 28]. Therefore, there is a need for continued research in this area to allow physicians to provide their patients with robust data on which to make informed decisions about whether to undergo RDN for the treatment of hypertension.

The current body of evidence for the efficacy and safety of RDN from sham-controlled clinical trials means that a recent European Society of Hypertension position paper has described RDN as an appropriate, evidence-based option for the treatment of hypertension [38]. In addition, guidance from Asian experts [39] and the Italian Society of Arterial Hypertension [40] notes that RDN has a role in the management of difficult-to-treat, resistant or uncontrolled hypertension, and that RDN could be considered as an earlier line of therapy rather than just a “last resort” option [39]. These recommendations provide good evidence-based guidance, but any decision about treatment options should be a shared process between the patient and their physician that also takes patient preference into account.

### Strengths and limitations

The key strength of this study is the large sample size that includes patients from all prefectures across Japan. However, the results have limited external validity because they are only applicable to the setting in which they were obtained (i.e. Japanese patients with hypertension). Furthermore, this was a self-reported internet survey, meaning that source verification was not performed and the sample may be non-representative; for example, there may have been fewer responses from patients with hypertension who were less familiar with the internet and older adults. Another important point to note is that the data in this study are relevant to the time the survey was conducted, and do not reflect any potential effects of subsequent publications showing the efficacy and tolerability of RDN, such as the RADIANCE-HTN TRIO study [14], on patient preference and physician recommendations. Finally, adherence was self reported and was not verified using a validated questionnaire (e.g. Morinsky Medication Adherence [MMAS-8]) or any objective measures.

## Conclusions

A relevant proportion of Japanese patients with hypertension expressed a preference for RDN, especially males, younger patients, those experiencing drug-related side effects or non-adherence, and in patients with higher BP levels or comorbid heart failure. Patient preference should be taken into account when making shared decisions about antihypertensive therapy, alongside BP values, circadian patterns of BP, the overall cardiovascular risk profile, and tolerability and adherence to drug treatment.

## Supplementary information

Supplementary Methods
