# Supplementary material for: A Japan nationwide web-based survey of patient preference for renal denervation for hypertension treatment
Source: Hypertens Res. 2021 Oct 17;45(2):232–40. doi: 10.1038/s41440-021-00760-9 (PMC8766287; doi:10.1038/s41440-021-00760-9)
Supplement: Supplementary file 1 — Supplementary Methods [file 41440_2021_760_MOESM1_ESM.pdf]

ONLINE SUPPLEMENT

A Japan nationwide web-based survey of patient preference for renal denervation for hypertension treatment

Kazuomi Kario[1], Hideaki Kagitani[2], Shoko Hayashi[2], Satsuki Hanamura[2], Keisuke Ozawa[2], Hiroshi Kanegae[1]

[1]Division of Cardiovascular Medicine, Department of Medicine, Jichi Medical University School of Medicine, Tochigi, Japan

[2]Clinical Development Department, Terumo Corporation, Tokyo, Japan

Supplementary Methods

Survey questionnaire

Q. Please provide your medical history.

Q. How often do you visit your doctor for hypertension treatment?

Q. Do you measure your blood pressure regularly?

Q. Please provide your most recent home and office blood pressure readings.

Q. Please report the number of medications prescribed, including antihypertensive drugs and others.

Q. Please list all classes of antihypertensive drugs that you have been prescribed.

Q. Are you prescribed combination drugs for hypertension treatment?

Q. Please report your height and weight.

Q. Which facility do you visit for hypertension treatment.

Q. How long does it take to get to the clinic/hospital for hypertension treatment?

Q. Do you work the night shift at least once per month?

Q. Please state how long you have had hypertension.

Q. Please state how long you have been taking antihypertensive drugs.

Q. Did you take all your antihypertensive drugs as prescribed in the last month?

Q. Please state the reason(s) why you forgot to take, or did not take, your antihypertensive drugs in the last month.

Q. Please report all the side effects that you have experienced since you started taking antihypertensive drugs.

Q. Did you change or discontinue prescribed antihypertensive drugs when you experienced these side effects?

Q. Please rate your satisfaction with hypertension treatment.

Q. What is the feedback on your blood pressure from your doctor?

Q. By how much would you want to lower your systolic blood pressure if one more antihypertensive drug was added?

Q. Please describe any concerns about your hypertension treatment.

Q. Please state what you want to achieve in your hypertension treatment.

Q. Please look at the attached document for details of renal denervation. Do you want to undergo renal denervation therapy?

Q. Assuming you have uncontrolled hypertension, would you prefer renal denervation or additional antihypertensive drugs?

Q. Please state the requirements for you to undergo renal denervation.

Q. By how much would you want to lower your systolic blood pressure if you underwent renal denervation?

Q. How many prescribed antihypertensive drugs would you like to stop taking if you underwent?

Q. What probability of reducing systolic blood pressure by 10 mmHg would be accept if undergoing renal denervation?

Q. Assuming you will undergo renal denervation, please state your concerns and questions on renal denervation.

Q. How do you inform yourself about hypertension treatment?

Q. Who influences your decisions relating to hypertension treatment?

Supplementary Table 1. Patient profiles in subgroups with and without home blood pressure data

Characteristic	Total (n=4,107)	Home BP measurements		p-value
		Yes (n=2,392)	No (n=1,715)	
Age, years	59.1±11.4	59.8±11.6	58.2±11.0	<0.001
Male, n (%)	2,756 (67.1)	1,577 (65.9)	1,179 (68.8)	0.058
Body mass index, kg/m^2	25.6±4.7	25.1±4.4	26.3±4.9	<0.001
Duration of hypertension, years	11.9±9.6	11.4±9.5	12.5±9.1	<0.001
**Office blood pressure**				
SBP, mmHg	135.3±13.6	135.7±14.2	134.7±12.7	0.022
DBP, mmHg	82.9±10.7	82.9±11.1	83.1±10.2	0.572
Uncontrolled SBP or DBP (≥130 or ≥80 mmHg), n (%)	3,397 (82.7)	1,964 (19.9)	1,433 (16.5)	0.006
Uncontrolled SBP or DBP (≥140 or ≥90 mmHg), n (%)	1,597 (38.9)	909 (57.9)	688 (59.9)	0.200
**Morning home blood pressure**				
SBP, mmHg	-	134.6±14.3	-	-
DBP, mmHg	-	83.1±11.5	-	-
Uncontrolled SBP or DBP (≥125 or ≥75 mmHg), n (%)	-	2,150 (89.9)	-	-
Uncontrolled SBP or DBP (≥135 or ≥85 mmHg), n (%)	-	1,364 (57.0)	-	-
**Medical history, n (%)**				
Diabetes mellitus	1,066 (26.0)	553 (23.1)	513 (29.9)	<0.001
Cardiovascular disease	775 (18.9)	476 (19.9)	299 (17.4)	0.046
ASCVD	707 (17.2)	442 (18.5)	265 (15.5)	0.011
CAD	412 (10.0)	250 (10.5)	162 (9.5)	0.290
Stroke	303 (7.4)	196 (8.2)	107 (6.2)	0.018
Aortic aneurysm/dissection, PAD	181 (4.4)	123 (5.1)	58 (3.4)	0.007
Heart failure	215 (5.2)	133 (5.6)	82 (4.8)	0.269
Chronic kidney disease	259 (6.3)	166 (6.9)	93 (5.4)	0.049
**Medical facility for hypertension treatment, n (%)**				
Medical university hospital	177 (4.3)	119 (5.0)	58 (3.4)	
Hospital	1,035 (25.2)	651 (27.2)	384 (22.4)	<0.001
Clinic	2,895 (70.5)	1,622 (67.8)	1,273 (74.2)	
**Antihypertensive therapy**				
Number of antihypertensives, n (%)				
1	1,595 (38.8)	886 (37.0)	709 (41.3)	
2	2,127 (51.8)	1,261 (52.7)	866 (50.5)	0.002
≥3	385 (9.4)	245 (10.2)	140 (8.2)	
Time on antihypertensives, years	10.8±8.6	10.3±8.8	11.5±8.3	<0.001
Poor adherence[#], n (%)	512 (12.5)	288 (12.0)	224 (13.1)	0.329
Side effects present[§], n (%)	1,424 (34.7)	911 (38.1)	513 (29.9)	<0.001

Values are mean ± standard deviation, or number of patients (%).

[#]Poor adherence was defined as missing at least one antihypertensive dose per week.

[§]Side effects attributable to antihypertensive medication.

ASCVD, atherosclerotic cardiovascular disease; CAD, coronary artery disease; DBP, diastolic blood pressure; PAD, peripheral artery disease; SBP, systolic blood pressure.

Supplementary Table 2. Effect of the prevalence and side effects of antihypertensive therapy on patient preference for renal denervation

Variable	N	Patient preference*, n (%)	Univariate analysis		Logistic regression analysis	
			Crude OR (95% CI)	Crude p-value	Adjusted OR (95% CI)	Adjusted p-value
**Dizziness**						
Yes	411	174 (42.3)	1.77 (1.42–2.20)	<0.001	1.29 (102–1.65)	0.038
No	1,981	581 (29.3)	reference	-	reference	-
**Frequent urination**						
Yes	252	116 (46.0)	2.00 (1.54–2.61)	<0.001	1.40 (1.05–1.87)	0.023
No	2,140	639 (29.9)	reference	-	reference	-
**Hypotension**						
Yes	192	68 (35.4)	1.21 (0.89–1.65)	0.231	*n.s.*	-
No	2,200	687 (31.2)	reference	-	reference	-
**Palpitation, tachycardia**						
Yes	161	82 (50.9)	2.40 (1.74–3.32)	<0.001	1.51 (1.05–2.16)	0.025
No	2,231	673 (30.2)	reference	-	reference	-
**Dry mouth**						
Yes	152	84 (55.3)	2.89 (2.07–4.03)	<0.001	2.01 (1.40–2.87)	<0.001
No	2,240	671 (30.0)	reference	-	reference	-
**Tiredness/weakness**						
Yes	131	62 (47.3)	2.03 (1.43–2.90)	<0.001	*n.s.*	-
No	2,261	693 (30.7)	reference	-	reference	-
**Constipation**						
Yes	110	53 (46.9)	1.98 (1.36–2.90)	<0.001	*n.s.*	-
No	2,279	702 (30.8)	reference	-	reference	-
**Edema**						
Yes	106	44 (40.0)	1.47 (1.00–2.18)	<0.001	*n.s.*	-
No	2,282	711 (31.2)	reference	-	reference	-
**Headache**						
Yes	106	59 (55.7)	2.87 (1.94–4.25)	<0.001	1.95 (1.28–2.97)	0.002
No	2,296	696 (30.5)	reference	-	reference	-
**Hot flashes**						
Yes	84	40 (47.6)	2.03 (1.31–3.14)	<0.001	*n.s.*	-

No	2,308	715 (31.0)	reference	-	reference	-
**Hot flashes (women only)**						
Yes	37	20 (54.1)	3.85 (1.98–7.51)	<0.001	3.07 (1.53–6.15)	0.002
No	778	182 (23.4)	reference	-	reference	-
**Persistent cough**						
Yes	73	27 (37.0)	1.28 (0.79–2.08)	0.311	*n.s.*	-
No	2,319	728 (31.4)	reference	-	reference	-
**Sexual dysfunction**						
Yes	48	29 (60.4)	3.40 (1.89–6.11)	<0.001	2.29 (1.24–4.22)	0.008
No	2,344	726 (31.0)	reference	-	reference	-
**Sexual dysfunction (men only)**						
Yes	44	27 (61.4)	3.04 (1.64–5.63)	<0.001	2.46 (1.30–4.66)	0.006
No	1,533	526 (34.3)	reference	-	reference	-
**Dermatitis**						
Yes	42	17 (40.5)	1.49 (0.80–2.77)	0.210	*n.s.*	-
No	2,350	738 (31.4)	reference	-	reference	-
**Confusion/difficulty thinking**						
Yes	23	15 (65.2)	4.13 (1.74–9.78)	0.001	*n.s.*	-
No	2,370	740 (31.2)	reference	-	reference	-
**Photosensitivity**						
Yes	22	9 (40.9)	1.51 (0.64–3.54)	0.343	*n.s.*	-
No	2,369	746 (31.5)	reference	-	reference	-
**Abnormal laboratory findings**						
Yes	22	11 (50.0)	2.19 (0.94–5.06)	0.062	*n.s.*	-
No	2,370	744 (31.4)	reference	-	reference	-
**Fall**						
Yes	14	9 (64.3)	3.94 (1.32–11.79)	0.008	*n.s.*	-
No	2,378	746 (31.4)	reference	-	reference	-
**Other**						
Yes	56	18 (32.1)	1.03 (0.58–1.81)	0.925	*n.s.*	-
No	2,336	737 (31.6)	reference	-	reference	-

CI, confidence interval; n/a, not applicable; *n.s.*, not significant; OR, odds ratio.

*Patient preference for renal denervation was defined as a survey answer of "I want to undergo renal denervation" or "I would rather undergo renal denervation".

Supplementary Figure 1. Overview of renal denervation for patients.

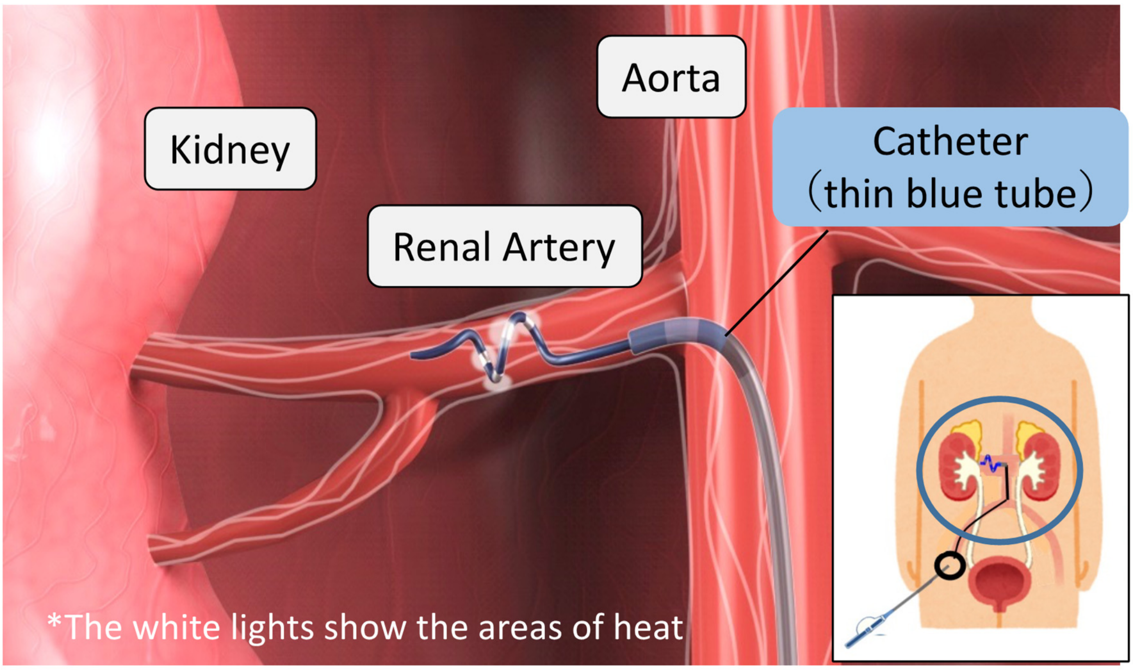

■**Procedure:** The procedure is performed using a flexible tube called a catheter. A catheter is inserted into an artery near your groin and guided to the renal artery. Then the tip of the catheter generates radiofrequency energy or ultrasound to heat the renal artery wall to reduce the activity of the over-active sympathetic nerves. **Since the nerves are one of the causes of hypertension, renal denervation is expected to lower blood pressure.**

■**Treatment time:** About an hour

■**Hospitalization period**: Overnight hospitalization

The renal denervation is in the research stage and has not been approved in Japan. However, some clinical trials are conducting in many countries, including Japan, toward the actual clinical use in several years.

Supplementary Figure 2. Sources of information used by patients to get information about hypertension and to inform their decision about use of renal denervation (RDN)

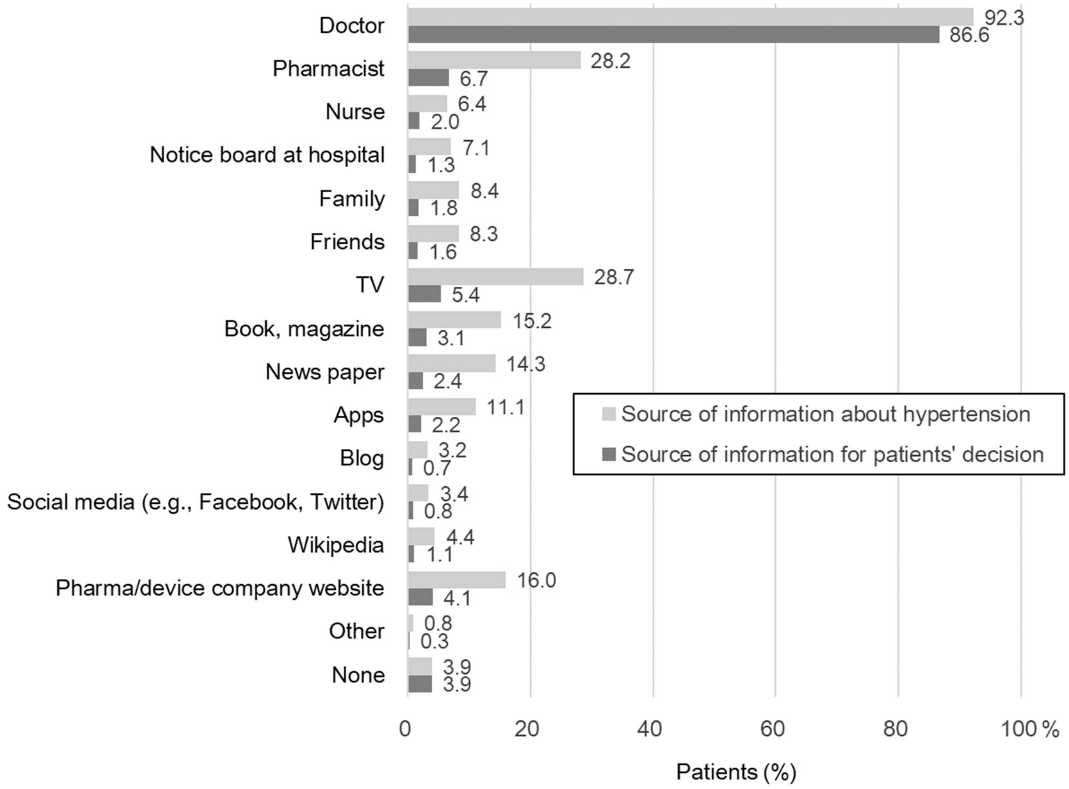

Supplementary Figure 3. Patient expectation of the reduction in blood pressure that they would achieve if treated with renal denervation (RDN) in the current study compared with a previous study conducted in Germany[1]

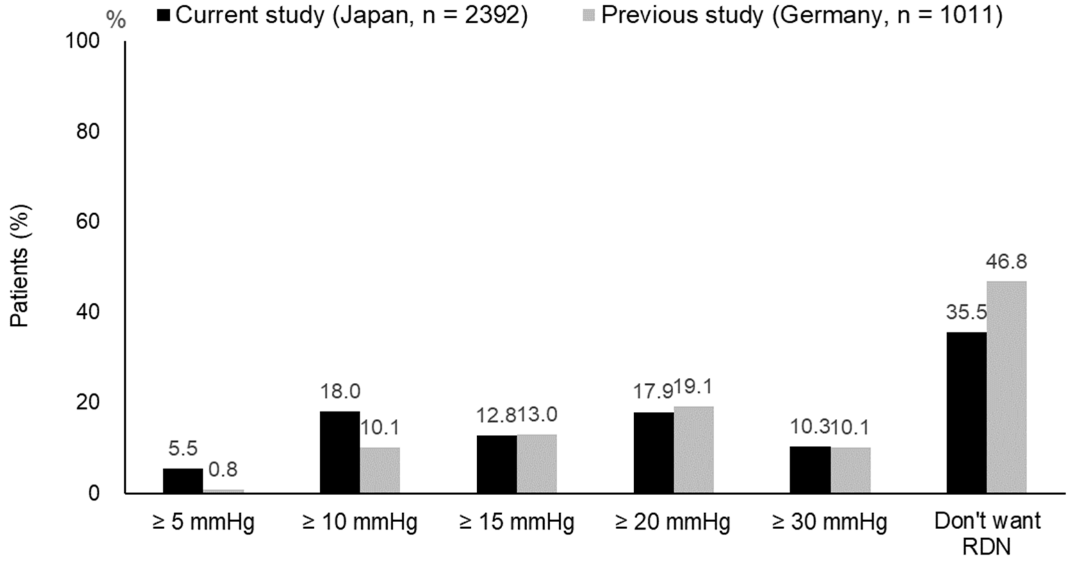

REFERENCES

1. Schmieder RE, Högerl K, Jung S, Bramlage P, Veelken R, Ott C. Patient preference for therapies in hypertension: a cross-sectional survey of German patients. *Clin Res Cardiol.* 2019;**108**(12):1331-1342.
